# Disabled people’s organisations and the disability movement: Perspectives from Burkina Faso

**DOI:** 10.4102/ajod.v8i0.500

**Published:** 2019-04-29

**Authors:** Lara Bezzina

**Affiliations:** 1 Private, Mosta, Malta

## Abstract

**Background:**

In Burkina Faso, the disability movement is rather weak, both in terms of funding and staffing – its range does not extend far outside the capital city and is largely dependent on international non-governmental organisations (INGOs). Despite the huge number of grassroots disabled people’s organisations (DPOs), many of these organisations do not function beyond the occasional meeting and celebration of the International Day of Persons with Disabilities. The reasons for this are various, including dependency on external funding (such as from international organisations), lack of access to resources, being dependent on voluntary members, and lack of organisation.

**Objectives:**

This article looks at the functioning of – and politics governing – DPOs in Burkina Faso, their significance in the lives of people with disabilities and the challenges they encounter.

**Method:**

This article is based on research findings obtained through interviews conducted with people with disabilities, as well as INGOs working with people with disabilities and state authorities in Burkina Faso.

**Results:**

Evidence suggests that the farther people with disabilities are from the capital, the lesser are their chances of being heard and of being involved in decision-making. However, DPOs offer a haven for many, offering people with disabilities solace in meeting other members and finding a sense of belonging in these associations. Others give importance to the role of DPOs in raising awareness and human rights advocacy.

**Conclusion:**

Finally, the article raises the question as to what the future of DPOs in Burkina Faso might entail.

**Keywords:**

Burkina Faso; disability identity; disability movement; disabled people’s organisations; income-generating activities; international non-governmental organisations; socialisation; *vie associative*; voluntary organisations; urban–rural divide.

## Introduction

The importance of collective power and self-organisation has been demonstrated through the achievements of the disability movement in western contexts, and has been documented by disability scholars (e.g. Oliver 1996; Shakespeare 1996). In Burkina Faso, however, the disability movement is rather weak in terms of staffing and funding. While it enjoys a certain level of political access and support in the capital, Ouagadougou, its reach beyond the city is minimal. Politics at the national federation level does nothing to solidify an already weak movement, and support which is presumed to be forthcoming for smaller disabled people’s organisations (DPOs) does not seem to exist. Rather than a disability ‘movement’ in the western sense, in Burkina Faso, there is a multitude of grassroots DPOs, some of whom are supported by international non-governmental organisations (INGOs). Further still, the terrain of self-organisation is uneven: while in most rural areas, collective organisation, activism and mobilisation of people with disabilities are still very nascent, in urban areas they have been able to organise through DPOs for a number of years. Paradoxically, while some people with disabilities are just becoming acquainted with self-organisation and perceive it as an advantageous and effective way to organise, others with greater experience of DPOs are becoming increasingly disenchanted with them. In both cases, self-organisation and collective power in Burkina Faso are not strong. Disabled people’s organisations are often ‘reinforced’ by INGOs, which are ‘necessary’ for DPOs’ functioning, but also keep the same DPOs dependent.

In what follows, I examine the grassroots level of the disability ‘movement’ in Burkina Faso, the different attitudes of people with disabilities towards DPOs and what motivates them to create and join DPOs, and what makes these DPOs work or otherwise. The article also looks at the role that DPOs play in the lives of people with disabilities and the challenges these DPOs are facing today.

### Burkina Faso

Burkina Faso, located in West Africa, is rated as a low-income country by the World Bank (2018a) and is ranked 185th (out of 188) countries on the Human Development Index (United Nations Development Programme 2016). The country is landlocked and highly dependent on agriculture, both of which hinder its economic development. External aid plays a significant role in Burkina Faso’s development, be it private (mostly nongovernmental organisations [NGOs]) or public, which comes largely from France (which colonised Burkina Faso until 1960) and the European Union (LAFB^1^ LAFB 2007).

Burkina Faso has 13 regions and 45 provinces. The regions are headed by a governor, while the provinces are headed by a high commissioner (Mahieu & Yilmaz 2010). The three major urban areas are Ouagadougou, Bobo-Dioulasso and Koudougou (Howorth 1999; Zongo 2004). Urbanisation in the large cities in Burkina Faso is taking place at a great speed, and factors such as overpopulation (which prevents people from improving their living conditions), young educated people desiring ‘a better life’ in the city, droughts and desertification all contribute to the rural exodus characterising Burkina Faso in recent years (LAFB 2007).

Issues of disability and development in Burkina Faso have so far been under-researched. Statistical information on people with disabilities in Burkina Faso is hard to come by (Handicap International^2^ [HI] 2005). A 2009 report issued by government entities states that 1.2% of the population of the country live with a disability (*Ministѐre de l’Economie et des Finances* [Ministry of Economy and Finance], *Comité National du Recensement* [National Censuses Committee] and *Bureau Central du Recensement* [Central Censuses Office] 2009). This seems to be in stark contrast to the figure of 15% of the world’s population reported by the World Bank (2018b) and the World Health Organization (WHO 2018). A director at the ministry responsible for people with disabilities in Burkina Faso (Email to author, 01 October 2015) explains this idiosyncrasy:

With regard to the figure of 1.2% of people with disabilities, one must note that these figures fall short of the reality on the ground. This could be due to the lack of knowledge on the notion of disability by the researchers, or the unfavourable social representations linked to disability, which often result in people not wanting to declare a disabled relative. There is the tendency to hide them.

Furthermore, the Swedish development cooperation (Sida 2012) also reports that the statistics collected by the National Institute of Statistics and Demography in Burkina Faso (and the ensuing figure of 1.2%) are not dependable, because efforts to collect in-depth statistics have not been impressive, and much of the statistics are collected in the more densely populated urban areas (rather than smaller rural ones).

## Methodology

This article is based on research conducted with adults with disabilities in Burkina Faso from June 2014 to June 2015. Over 300 interviews (see Table 1) were conducted with people with physical, sensory and intellectual disabilities; grassroots and umbrella DPOs^3^; INGOs working in the disability domain; as well as state authorities. Apart from the formal, semi-structured interviews, there were countless informal conversations that were indispensable to the research, not only in elucidating otherwise unclear data and providing context, but also in triangulating data obtained from formal interviews.^4^

**TABLE 1:** Interviews held in Burkina Faso.

Entity	Region	No. of interviews	Total no. of interviews
Individual people with disabilities	Est	47	228
	Centre	30	
	Plateau-Central	4	
	Cascades	146	
	Hauts-Bassins	1	
DPOs	Est	10	65
	Centre	33	
	Plateau-Central	1	
	Cascades	17	
	Hauts-Bassins	4	
Umbrella DPOs	Centre	9	9
INGO field offices	Centre	8	10
	Cascades	1	
	Email Exchange	1	
Authorities	Centre	3	15
	Cascades	12	

Note: Total number if interviews = 327.

DPOs, disabled people’s organisations; INGO, international non-governmental organisation.

The majority of interviews were conducted in three regions: the Centre region (the central region that consists mostly of the capital city, Ouagadougou), the Est region (the Eastern region) and the Cascades region (the South-westernmost region). These three regions were chosen as being the farthermost regions of the country from East to West, and covered the southern, western, central and eastern regions of Burkina Faso. Furthermore, the Est and Cascades regions provide a contrast to the urban capital as well as an urban–rural contrast within themselves, while the Centre region was also included because of its high density of DPOs.

## Research findings

### Disabled people’s organisations

Statistics on the exact number of DPOs in Burkina Faso are scarce. Handicap International (HI 2010) reports that there are approximately 25 grassroots DPOs in the Est region and approximately 99 in the city of Ouagadougou. I use the word ‘approximately’ because these DPOs are a mix of organisations. Firstly, although they are referred to as organisations^5^ in general, most of them are associations, and are called so in the individual DPOs’ names. Secondly, the DPOs’ functions, activities and sizes vary considerably. In rural areas, each municipality tends to have its own DPO, which encompasses people with all kinds of disabilities. These municipal DPOs are then members of a provincial coordination of DPOs, which are, in turn, members of a regional coordination. These regional coordinations are then members of one of the national federations of DPOs, the *Fédération Burkinabè des Associations pour la Promotion des Personnes Handicapées* (FEBAH): Burkinabe Federation of Organisations for the Promotion of Disabled People or *Réseau Nationale des Organisations des Personnes Handicapées* (ReNOH): National Network of Disabled People’s Organisations. The FEBAH had been the only national federation until 2012, when, following internal disputes, another national federation, the ReNOH, was created. The latter has a much smaller reach outside the capital and a much smaller membership than the FEBAH.

In urban areas such as Ouagadougou and Bobo-Dioulasso (the second largest city), the DPOs are more varied. There are DPOs whose membership is limited to people with specific disabilities, or women with disabilities. There are others that are more like cooperatives, such as an organisation of artisans with disabilities coming together to produce and sell. Many grassroots DPOs, especially in Ouagadougou, were created more as profit-making organisations, or at least with the aim of generating profit. In the larger urban areas, one also finds DPOs that have schools for children with disabilities (and sometimes without disabilities), as well as a few organisations (generally of parents) of children with specific intellectual disabilities, such as autism. Another category of DPOs – which for clarity’s sake will be referred to here as umbrella DPOs – comprise grassroots DPOs, usually ones pertaining to the same disability, gender or activity. For example, an umbrella DPO might be made up of grassroots DPOs of people with visual disabilities, of sports DPOs or of DPOs of women with disabilities. These umbrella DPOs, generally based in Ouagadougou, are also members of the national federations of DPOs. This grouping of small DPOs into larger umbrella DPOs is favoured by INGOs who intervene in the disability domain in Burkina Faso. For example, the International Programmes Director of an INGO^6^ explains that donors:

‘will want to focus in one area…. They will not want to fund a physical disability organisation here, a visually impaired organisation here, an auditory impaired… and they will want to focus it in one place that can cover all.’

This ‘decentralisation’ of DPO structures has its positive aspects in that there are grassroots DPOs at municipal level, comprising people with disabilities from the villages of that municipality. Nonetheless, it also keeps the same people with disabilities from rural areas at bay, because there are usually DPOs at higher ‘levels’ representing them. Thus, when, for instance, the FEBAH calls people with disabilities for a meeting in Ouagadougou, it would generally be the leaders of the regional coordinations who attend. People with disabilities from the farthest rural areas are rarely involved in national events. As can be expected, DPOs in urban areas, especially those in Ouagadougou, have more access to resources (mainly INGO funding) and opportunities (such as training). They are also the primary beneficiaries of government donations such as wheelchairs and three-wheel motorcycles. Rural DPOs generally encounter difficulties in all aspects of organisation functioning, including: bringing together members from far flung villages, having low numbers of literate members, having less or no money for functioning and possessing no building where to meet. In both urban and rural areas, however, there are a great number of non-functioning DPOs. There are several reasons for this, including the fact that, as will be discussed further on, most DPOs are made up of members working on a voluntary basis and thus have other – more immediate – priorities in their lives.

Another significant factor is that many members join DPOs to benefit from aid. In their 2002 annual review, Action on Disability and Development (ADD) reported that memberships of DPOs in Burkina Faso increased by 21% in that year (Albrecht 2006). The proliferation of INGOs led to ‘the emergence of a new breed of indigenous NGOs’ owing to the fact that INGOs need ‘partners through whom to implement their projects, so in some cases they were instrumental in creating local organisations for the purpose’ (Sharp 1990:40). Many DPOs in Burkina Faso were created at the behest of INGOs, who prefer to work with groups, rather than with individual people. This was the case in the Est region of the country where an INGO used to work. In the Comoé province in the Cascades region, where another INGO was intervening, the INGO helped create the provincial coordination made up of DPOs in the municipalities of the same province. Thus, many DPOs today are created in the hope of receiving aid from INGOs, as will be discussed shortly. This being said, DPOs play beneficial roles in the lives of people with disabilities.

### The benefits of disabled people’s organisations

Becker (1980:68) states that ‘[*v*]oluntary associations are based on common interests… [*t*]hey provide social nurturance to their members’. She observes that:

When the individual is continually reminded of his or her variance from others this increases the level of stress…. Stress can best be minimized by playing down the overt differences of the disability and thus its importance. Among a tightly knit reference group… the problems of coping with the disability are forgotten or dealt with by joking. (Becker 1980:78)

While in this example, Becker is describing a deaf community, her comments are applicable to groupings of people with other types of disability, as well as to the Burkinabe context and the question of the usefulness of DPOs to their members. At the Centre of people with disabilities in Ouagadougou, people with physical disabilities meet up to work, socialise, eat and drink. People with disabilities joke with each other, calling each other ‘hey you, disabled’ and making jokes that they contaminated each other with disability, or that they wanted to become disabled like each other. Disabled people’s organisations are a source of identification and socialisation for many people with disabilities, thus also serving as a haven and offering a sense of solidarity:

‘The DPO was created in [*19*]97… to raise awareness… to form a group, an organisation, so [*we*] can be heard by the population, by the authorities of the municipality…. The objective of the organisation was first of all to create a setting so that all disabled people can meet up to share ideas, their joys, their worries and their sorrows; because by staying at home, alone, you are isolated: you are sitting around, you don’t go out, you are lost. So the aim was to get disabled people to come out and group them, train them, educate them and integrate them in society.’ (Interview with a rural DPO Executive Committee)

From a study she conducted in North America, Becker (1980:98) observes that a group of deaf people:

defined by the larger society as afflicted, have created a small society that has had an influence throughout life on… their disability…. Individuals in this specially created society have used group membership to achieve a nonstigmatized personal identity and normalized social relationships.

Similarly, in Burkina Faso, the research participants talk of how they find solidarity in DPOs and the comfort of knowing that there are other people who, like them, have a disability and who go through similar experiences. Some go as far as to say that they do not feel disabled when they are among other people with disabilities, as the following reveals:

‘[*I became a member of the DPO*] because there, at least, you are with disabled people: when you see them, you [*see*] … that you too are a disabled person…. Because before … I did not go out, I stayed at home. I put it in my head that only I am disabled. But since I became part of the DPO, I see that it’s not only me who suffers^7^ from disability, there are many people who suffer from their disability.’ (Interview with Josiane,^8^ woman with physical disability, age 31^9^)

‘[*Disabled people’s sport*] … gives me health and… if you are alone, it’s dull, but when you go to disabled people’s sport, you find… that there’s a nice atmosphere, well, you don’t even remember you are disabled, so you feel at ease …’ (Interview with Salif, man with physical disability, age 29)

In Burkina Faso, where there are people with disabilities who did not know that there are other people with disabilities like them before they joined a DPO, the sense of identity emerging from belonging to a group of people with disabilities takes on an accentuated significance:

‘When I arrived [*at Noong Taaba*^10^]… I was happy, because… when I was alone, I thought I was the only blind person. Once I had been there [*to Noong Taaba*], there were a lot of things which consoled me: I saw that I was not alone.’ (Interview with Jean, man with visual disability, age 55)

The importance of these smaller ‘created societies’ in the life and identity of a person with disability is further demonstrated by Murphy et al.’s (1988) observation that, unlike other stigmatised groups (such as those discriminated against on the basis of racial or religious grounds), most people with disabilities are not brought up by parents with disabilities, nor do they grow up amongst people with disabilities. Thus, people with disabilities are not ‘socialised’ in the same way that, for example, a person from a religious minority would have been, and so would not have learnt ‘how to deal with a sometimes hostile world’ (Murphy et al. 1988:241). Furthermore, many people with disabilities become so through accident or illness and acquire the impairment when they are already adults. Yet, even those who are born with a disability, or become disabled when very young, are ‘usually brought up by parents who have no knowledge of the social problems of the disabled’ (Murphy et al. 1988:241). Referring to institutions such as special schools and rehabilitation centres, Murphy et al. (1988:241) observe that:

In a way, the physically handicapped have to be ‘resocialized’…. And the school or hospital environments may become so comfortably familiar that they are preferred to home.

However, people with disabilities in Burkina Faso who are adults today rarely had the opportunity to go to schools where they could mingle with other children with disabilities, if they went to school at all. Disabled people’s organisations therefore replace these ‘socialisation’ institutions:

‘I have been coming to the Centre [*of people with disabilities*] since 2004. I started coming because I learnt that there is an organisation of people of my kind, that is, people like me, people with a visual disability… and on that day I was *very* happy because I heard that there were people who were like me, and that I can join the organisation. I was very happy; I was in a hurry, even, to come and meet them.’ (Interview with Rachid, man with visual disability, age 28)

In these examples, DPOs become a microcosm of society where people with disabilities experience a non-stigmatised identity and socialise with people with similar (or other) disabilities.

Disabled people’s organisations can also be a source of awareness raising and advice:

‘Thanks to the organisation… [*we*] learnt different techniques to protect [*our*]selves… [*from the sun*]: to wear long sleeves… to apply the [*sun protection cream*] they give [*us*] when [*we*] are in the sun; so, it helps [*me*] a lot. … [*I had gone*] to the pharmacies and I couldn’t find [*the cream*], and it was thanks to the organisation that [*I*] saw the product and started using it.’ (Interview with Djibril, man with albinism, age 29)

Furthermore, DPOs have the potential to ‘represent… the space where subaltern, hitherto inaudible and unarticulated views can be expressed’ (Tandon 2003:65). Disabled people’s organisations are one of the spaces where people with disabilities, whose views are often not heard by the dominant hegemonic society, can be heard. A university student articulates the importance of collective mobilising:

‘When we come to the university here… I think… it’s almost a duty… to take part and participate… in the organisation… to campaign in the organisation…. It’s better to find oneself in a community, in a group: like that we can campaign together and it gives us more strength; we can claim our rights.… As we say, there is strength in unity…. Moreover, here we have practically the same realities… the same problems, so why not unite ourselves…?’ (Interview with Aboubacar, man with visual disability, age 26)

Nonetheless, as mentioned previously, despite the multitude of DPOs in Burkina Faso, fully-fledged organisations that play a role in advocacy, lobbying or awareness-raising are few and far between.

### The challenges encountered by disabled people’s organisations

One reason for the lack of a cohesive and strong disability movement is that the majority of DPOs are voluntary organisations; thus, the members evidently give priority to their personal income-generating activities^11^:

‘Before, every twenty-one days [*we*] met to talk among [*our*]selves and all that. But… the president has his own work; and the… [*General*] Secretary… it was her, before, who mobilised the people, and the people used to meet. But now she, too, has gained an [*income-generating*] activity for herself, so she does not have time anymore to [*gather*] the people, and so she does not make the effort to bring people together.’ (Interview with Aida, woman with visual disability, age 36)

The major reason for the lack of DPO functioning, however, is explained in the following excerpt:

LB^12^: ‘What do you think is needed in Burkina so that the disabled person is able to integrate in society?’

J: ‘There needs to be a change in mentality.’

LB: ‘But how? Who is going to change the mentality?’

J: ‘The organisations of disabled people…’

LB: ‘Do you think that these organisations are… attaining their goals?’

J: ‘Well, attaining their goals is a bit complicated, because… it’s the start of a beginning… and, moreover… the organisations have not understood why one creates an organisation: they [do] not have the organisational spirit.’

(Interview with Joseph, man with physical disability, age 55)

The ‘organisational spirit’, or *vie associative*, that Joseph refers to, is what makes up the life of an organisation: the role and contribution of the members, the functioning of the organisation and its work towards achieving its objectives. This is the significant factor that, according to Joseph, many DPOs lack, and which leads to numerous problems of self-organisation among people with disabilities. One province high commissioner observes that when people with disabilities come to her, it is always to ‘ask, ask, ask’, but they never show anything that they do. According to her, people with disabilities need to sit down and plan the way forward. Disabled people’s organisations, like many other grassroots organisations in Burkina Faso, have no activities apart from sitting around and waiting for financial support. The high commissioner asserts, however, that:

‘No one has “nothing”. You have to give something in order to receive. If someone gives all the time, he will get tired, but if it’s dynamic, the relationship won’t end… everyone has something to give.’

A similar observation is made by an INGO national director:

‘Even to gather for a General Assembly, they [*DPOs*] will ask an NGO for the financial means; yet an organisation shouldn’t be like this: [*by means of*] the membership fees, [*and*] donations from other people… the organisation should at least be able to meet to discuss its… common interests.’

The director’s comments also relate to how DPOs fall into the tendency of depending on external partners for funds, rather than attempting to generate funds internally. This observation touches upon the argument made by Kajimbwa (2006) that when INGOs implement their own programmes, it is likely that the beneficiary of the INGO will have a decreased sense of ownership and potential to act. Meanwhile, the same high commissioner makes a related observation on what is possibly hindering the functioning of DPOs:

‘People have associated the organisation with money…. [*However*,] it’s not money which enables you to live, but good practices which enable the money to stay.’

The high commissioner is talking of the large number of grassroots organisations (including DPOs) that are created for the sole aim of receiving aid or to access funding in general, a point that is reiterated by the president of a DPO:

‘[*We*] have an Executive Committee: a president, a general secretary, a treasurer, but it doesn’t function, because people have not understood, for a start, the interests of the organisation…. For them, when one says ‘the organisation’, people think that it’s to call them and give them money.’

Joining a DPO for economic reasons is not necessarily negative:

LB: ‘Are you a member of a disabled people’s organisation?’

F: ‘Yes…’

LB: ‘Which one?’

F: ‘… [the] national federation of artisans… of disabled people…’

LB: ‘And why did you become member of this organisation?’

F: ‘Because… since I do hairdressing… I am an artisan too. So, I am a part [of it] because… when you work alone it’s not good, but when you are in a group, it’s better… for example, if there is a call for products or services… and if you can do it, they give you the work.’

(Interview with Florence, physically disabled woman, age 37)

However, Florence already has her own income-generating activity, and only joined the organisation to strengthen her work, rather than to simply access funds. Furthermore, she later specifies: ‘each one of us works separately’ (Interview with Florence, physically disabled woman, age 37), a statement that captures one of the challenges that DPOs encounter, that is, that many of the participants in this research prefer to work on their own. Conversely, INGOs generally do not work with individuals, but with organisations, leading to the creation of numerous DPOs whose sole existence is to access external partners’ financial aid and support:

LB: ‘What was the reason for which you created the organisation?’

J: ‘… The reason for which I created the organisation: … I approached many people who told me “If you have an organisation, we can help you; but if you are on your own, we cannot help you”.’

LB: ‘Who said this?’

J: ‘… [*INGOs*]…: if you create an organisation and approach them, if your dossier is good, they will finance you…. So… it’s for this reason that I thought of creating the organisation.’

(Interview with Jean, man with visual disability, age 55)

The expectation for INGOs to work with DPOs seems to be present from the perspective of both DPOs *and* INGOs. For example, one of the reasons for the importance of unity in the DPO movement, according to the national director of an INGO, is attracting partners (such as INGOs) to work with them:

‘I think that for DPOs, there must be… unity… cohesion, because when we have an organisation that groups together all the DPOs… it’s even stronger:… when… there is one structure that coordinates all this, it gives them strength…. Even with the partners, when [*we*] feel that there is one structure… [*we*] can help [*them*]; but when it’s [*divided*] … it’s two, it’s three, each one fighting for their school of thought, it’s very difficult.’

Rather than uniting DPOs, however, the expectation of being funded by INGOs seems to have given rise to the *proliferation* of DPOs. Hence, much in the same way that the international aid system has become ‘increasingly dysfunctional’ and ‘has led to a system that is fragmented and duplicative, and places too heavy a burden on aid-receiving countries’ (Woods 2008:1218), INGO intervention has precipitated the creation of many DPOs in order for these to access foreign funding.

This ‘awaiting aid’ phenomenon then creates problems of functioning, as a regional DPO coordinator remarks:

‘There isn’t anyone who has taken the initiative to create [*an organisation*] and make it function… because… in this region there is the idea that when one creates an organisation, there will be [*financial*] support. But what if there is no such opportunity? People create and then they wait… There isn’t an organisation which has a clear policy which says ‘we will do this, we will do that.’

The idea of joining or creating a DPO to attract funds subsequently leads to members becoming discouraged over time. Numerous DPOs comment on the fact that meetings are no longer held because of the fact that the members feel that they are not gaining anything. This is further compounded if the DPO has already worked with an INGO in the past and thus enjoyed financial support for its activities. The loss of these benefits is felt more acutely by the members, who then refuse to attend meetings if there is no financial support:

‘With the partners [*INGOs*], people got used to having food, and so on, when there is a meeting…, so [*now*] we cannot… organis[*e*] big meetings, and so on.’

(Interview with a regional DPO coordinator)

Interventions by INGOs have conditioned many people with disabilities in Burkina Faso into expecting certain standards that are not possible after the INGO terminates its collaboration, mostly because DPOs do not possess as much financial capacity as INGOs. Many DPOs then cease to function when INGOs terminate their funding and collaboration. The phenomenon of grassroots organisations disintegrating once external support ends is not specific to disability. Atampugre (1997:62), writing about INGOs and grassroots development in Burkina Faso, comments on:

the extent to which groups quickly form in order to take advantage of opportunities in their external environment, disintegrating as soon as that objective has been met. It shows too that credit or financial support does not necessarily facilitate organisational development. On the contrary, it can undermine the ability of rural communities to organise in order to solve their own problems.

Dependency on external funding thus leads to the inactivity displayed by many DPOs in the interviews conducted for this research, as exemplified by the following DPOs:

S: ‘When we had the money, we went to the villages to raise awareness among the population, for example… the traditional chiefs, the religious chiefs, civil servants… so they support disabled people everywhere…’

LB: ‘When you say “when we had the money”, what does that mean? …’

E: ‘[*An INGO*]… came to help us with financing: when we had this, we did awareness raising in the villages.’

LB: ‘And now you don’t have the financing of [*the INGO*]… anymore?’

E: ‘No.’

LB: ‘So how do you do the awareness raising now?’

S: ‘At present we have almost stopped the plan, because we have nothing with which to travel. Today, it’s the money that counts: if you don’t have the money… to travel with a bicycle it’s complicated; if you have a motorbike, you can put petrol, if you have money; but if you have nothing, what will you do? Without money…’

(Interview with a DPO president and general secretary)

‘At that time, apart from the different quarterly meetings… there was nothing that disabled people did to promote… their autonomy. We were quite idle and… were waiting for [*a particular INGO*]… to come to our rescue.’

(Interview with a DPO president)

Today, the DPO which the latter president is talking about is once again doing nothing, after a period of intervention by two INGOs. The dependency of DPOs is thus clearly problematic for the long-term sustainability of disability activism and advocacy in Burkina Faso.

Apart from the lack of knowledge of organisational functioning, and the related notion of creating and joining a DPO as a means of accessing aid, there is the related problem of misappropriation of funds, which is also a nationwide issue. In the context of DPOs, the high commissioner of a province observes that when the organisation *does* access funds, it is then spent all at once, or simply ‘disappears’, and thus:

‘If you have a tree and keep cutting its branches, the trunk, you will end up with nothing. Even the roots will die.’ (Interview with province high commissioner)

The misappropriation of funds is a problem that pervades many DPOs in Burkina Faso. The point is also made by an INGO national director:

‘They are always waiting… they come to see an INGO, saying “this is our plan of action, we want to do this”. But when they are financed… the problem of governance proves to be a problem: often, we don’t know how the funds were spent.’

This is one of the major reasons that have led many people with disabilities, especially in urban areas, to become disillusioned with collective organisation through DPOs:

LB: ‘Are you a member of a DPO?’

C: ‘No.’

LB: ‘And why not?’

C: ‘… I was, before. But I left.’

LB: ‘Why? …’

C: ‘… things weren’t going well, [and] I resigned.’

LB: ‘Why? …’

C: ‘Things weren’t transparent… there… I prefer staying in my workshop.’

(Interview with Christian, man with physical disability, age 38)

The issue of misappropriation of funds (together with organisational functioning and the reasons underlying DPO creation) is also tightly linked with leadership, and the (lack of) transparency issues that Christian mentions. Underlying these structural drawbacks are the general hurdles encountered by people with disabilities in Burkina Faso, one of the major difficulties being the lack of education. The lack of access to schooling for many people with disabilities when they were young resulted in many adults today lacking the writing and reading skills necessary to lead and manage an organisation. This absence is felt more strongly when the DPO is working with INGOs, who often require reports and other written material (Mawdsley et al. 2002, 2005). This often gives rise to a situation where the DPO leaders are those who possess a certain level of education, but are not necessarily the ones who have the DPO’s and its members’ interests at heart. Unfortunately, these leaders tend to form an ‘elite’ group whose members are re-elected in consecutive elections, simply rotating roles from election to election. Fatou, a woman with physical disability, brings to life the issue of elite capture and other problems regarding DPO functioning in Burkina Faso (see Box 1).^13^

BOX 1 Fatou and the two disabled people’s organisations.

This evening, Fatou came to the Centre of people with disabilities to talk to me…. She was telling me how Carole, the supposed president of the disabled women’s organisation, has the key to the office where the material for making soap and soumbala is. Carole doesn’t come to the Centre anymore, and so the women cannot work. Fatou says that the women used to come to work but when they used to sell the soap they did not see the profits, they did not know where the money had gone! So they gave up and don’t come here to work anymore.

Fatou also says that she was not informed of the physically disabled people’s DPO meeting a couple of weeks ago, nor that it has been postponed to this Friday. Last time, the meeting was cancelled because Hamidou didn’t show up. (Hamidou was elected president in the last election; but on paper it still shows that Sayouba, the previous president, is president. Hamidou, who was president before Sayouba, is now president again. Rumour has it that he is avoiding coming to the meetings so that they will not renew the Executive Committee and change his position.) I asked Fatou why they keep electing Hamidou and she replies ‘Who is there to elect apart from him? Inoussa is busy with the workshop…’. I ask why not her. She says that Hamidou would make trouble for her if she proposes herself.

Note: Information derived from author’s field notes.

Fatou’s observations on the organisation of women with disabilities highlight not only the mismanagement of the same DPO and its funds, but also the fact that the president, Carole, has absolute power over what happens. They also explain why DPO members give up on being active in the organisation when funds are misappropriated, especially when it involves an income-generating activity from which the members should be profiting financially. Fatou’s observations also illustrate the leadership problems of another organisation of which she is a member: an organisation of people with physical disabilities, whose current president avoids the Executive Committee elections so that he remains president for as long as possible. Hamidou, the current president, was also the president two terms previously. Furthermore, should Fatou put herself forward as a candidate for president, the incumbent would make life difficult for her. There are not many other candidates who are eligible, that is, who possess the required level of education. Inoussa, whom Fatou mentions, is an educated member and possible candidate, but he is busy with other commitments (working in the metal workshop [which belongs to the DPO itself], preferring to dedicate his time to an income-generating activity). Finally, Fatou is not aware of the DPO meetings taking place, suggesting a lack of communication and information-relaying between DPO members. Similarly, Roland, a man with physical disability, says that he was not aware that the person accompanying us^13^ to his (Roland’s) home for interviewing is the current DPO president:

‘[*We*] haven’t made any renewals. To [*my*] knowledge, there haven’t been any renewals of the Executive Committee in which [*I*] participated… [*I*] was the president and [*my*] deputy was a visually disabled person…. Neither [*I*] nor [*my*] deputy… know that there is a [*new*] president, because [*for this to happen*] people must be present to say “we are going to elect a new committee, so that one became president, that one became…”’

The fact that many DPOs are led by the same people, electoral term after electoral term, and that elections are often not held, is also highlighted by INGOs:

‘There are always the same people at the head: there are no general assemblies.’ (Interview with an INGO national director)

Another noteworthy factor playing a role in DPO politics in Burkina Faso is related to gender. While Fatou is a member of both the women’s and the people with physical disabilities’ organisations in the small town where she lives, in more urbanised centres like Ouagadougou, the fact that most DPO presidents are male has led some women to break away and create DPOs of their own. Abigael, the president of a disabled women’s organisation in Ouagadougou, says that the organisation’s members used to form part of a larger organisation, but, as ‘women come second to men’, they decided to branch out and form their own DPO. Binta, the president of another women’s DPO in Ouagadougou, relates a similar story:

‘At first, we had a mixed organisation… [*and*] we thought… why don’t we, the women, separately… create our own organisation? Because, often… in the organisations, women don’t have decision-making roles. Moreover, in the [*executive*] committees, [*women*] hold posts… which do not have priority: often they are posts relating to women’s issues and such… So, in decision-making… men are in the forefront every time. Thus, together we reflected: why not create our own organisation? Because… it’s true, disabled people have problems; but… women have more problems than men… We have problems in our own right, so why not… see how we can overcome them?’

When asked to elaborate about the problems faced by women with disabilities, and how they differ to men’s problems, Binta continues:

‘Already, in our families, there are barriers between us, because, firstly, you have to sensitise the family… if you are accepted, it’s already something. If you are not accepted, this is already a problem. So, together, we have to reflect on all this. And then, we have children. And children are problems: a child always has a father, but… everything falls on the mother. So she has to seek work to… meet her child’s needs.’

Furthermore, as the government gives more importance to women’s issues than to disability issues, women with disabilities have also felt the need to create women’s DPOs (even if, like Fatou, they are still members of other DPOs), in order to be more visible. The ex-president of a women’s DPO explains:

‘[*We*] noticed that now, here in Burkina… the associations of women are more listened to. The authorities have put an emphasis on… associations of women. So that’s why [*we*] decided to… create [*our*] organisation.’ (Interview with Samira, physically disabled woman, age 51)

Gender concerns are not the only reason people branch out into new DPOs. Rather than coming together into a united disability movement, people with disabilities seem to be separating and following their own paths. Adama recounts:

‘[*I*] was the president of the coordination of disabled people… [*and*] since… for the moment… the coordination has stopped [*functioning*]… [*I created my own DPO*].’

Thus, despite all the hurdles discussed thus far, DPOs continue to proliferate. Nonetheless, the DPOs run by Binta, Abigael and Adama are based in Ouagadougou, with relatively good access to resources to create and sustain an organisation. Women (like people with disabilities in general) who live in the capital generally also have a higher level of education and more opportunities to branch out on their own. Furthermore, the constant mushrooming and branching out of DPOs seems to be more of an urban phenomenon than a rural one. In rural areas, people with disabilities seem to be at an earlier stage of self-organising, which also means that they are more marginalised in terms of being able to access support and assistance, or to articulate their needs. This can be seen, for example, in two rural municipalities in different provinces (see Boxes 2 and 3).

BOX 2 Creation of a rural disabled people’s organisation 1.

Upon arrival in the village, a guy who… is the brother of a disabled person came to meet us and brought us to a place under the mango trees where about 13 people are gathered…. Apparently they don’t really have a DPO in place but… they have been trying to put one in place.

Note: Information derived from author’s field notes.

DPO, disabled people’s organisation

BOX 3 Creation of a rural disabled people’s organisation 2.

Just arrived in the village…. [*S*]o today there was supposed to be the General Assembly (GA) which puts the DPO in place in this village, but since the guy in charge had been in Côte d’Ivoire, and the informer… failed to inform people, the GA has been postponed.

Note: Information derived from author’s field notes.

DPO, disabled people’s organisation.

An additional problem for DPOs is that umbrella organisations, which face similar leadership, functioning and dependency obstacles as the smaller DPOs, do not provide sufficient support to enable DPO mobilisation. At an umbrella DPO level, there is also the phenomenon of multiple leadership posts, that is, one person being the president of three different umbrella DPOs, as was the case at the time the fieldwork was being carried out. Meanwhile, the national federation of DPOs is riven by politics:

LB: ‘Can we speak of… a disabled people’s movement in Burkina?’

J: ‘It exists, but it functions very weakly…’

LB: ‘When you say it exists, it’s who?’

J: ‘It’s the two structures: ReNOH and FEBAH…but their actions are not translated on the ground…. The difficulty is the weak engagement of DPOs: they have a very weak engagement concerning the implementation of their rights… not to speak of the synergy… between the two organisations…. Having two federations… does this help us? I don’t think so.’

(Interview with Joseph, physically disabled man, age 55)

Joseph speaks of the two national federations of DPOs. The original federation was split into two (FEBAH and ReNOH), following disagreements. According to Joseph, there now needs to be a confederation to join these two federations and bring some unity to the disability movement. The problem, once again, is a problem of leadership:

‘It’s a problem of leadership: with white people, things are clear: you have done your mandate, you leave your place… [*for someone else. But here,*] some people have finished and don’t want to let go! They modify the statute; they create an Executive Secretariat which has even more power than the president! (Interview with Joseph, man with physical disability, age 55)’

‘I cannot say that the DPOs work well…. You know that usually DPOs have a problem of leadership…. Besides the leadership problem, there is also… the notion of organisation per se: it’s not yet well perceived, because they always put forth the problem of means, of lack of means…. Even when you look at DPOs which are well structured, there are always difficulties…. When you take the case of FEBAH, you see how it went: there is ReNOH, you have two federations…. They themselves don’t foster… cohesion…, because it’s always problems of leadership, internal power struggles, low blows.’ (Interview with an INGO national director)

The proliferation of DPOs and their lack of strength, whether they are rural- or urban-based, are major contributing factors, therefore, to the weak disability movement in Burkina Faso. This is then compounded by the lack of cohesion as well as leadership issues amongst DPOs, as commented on by an INGO national director:

‘I think the first thing that disabled people and their organisations should deal with, is the issue of organisation: the DPOs in Burkina are not organised…. They do not manage to get on with each other…. They do not really have an interest in uniting and… working in the same direction… It’s a question of organisation and also of governance… in the sense that it’s always the same disabled people who are at the head of the same DPOs. If I’m not happy here, I go to the other side… if I am not in the head, I leave and create my own… [*group*]. So we have a lot of organisations which exist. But what do they do? Nothing!’

## Conclusions

This article has explored the currents that underlie and influence DPOs in Burkina Faso, their creation, their functioning and the interplay between DPOs and INGOs. The difficulties encountered by DPOs are partly because of interventions by INGOs, which render DPOs dependent on foreign funding and support. Nonetheless, the argument here is not that there is no need for DPOs. Disabled people’s organisations provide a space where people with disabilities meet, whether or not they have similar disabilities. They provide a space in which comfort and solidarity are provided, and where ideas are exchanged. In addition, there is also a case for strength in numbers, which is important for those most marginalised and rendered invisible in society. Although the economic, political and social situation in Burkina Faso might present difficulties for many Burkinabes, people with disabilities need to be recognised in their own right. The DPOs provide an important space in which they have the possibility and opportunity to come together and speak out and fight for what is rightfully theirs. The challenge for Burkinabe DPOs is to become sustainable with minimal support from INGOs, and to become organisations that truly serve their members’ needs in working towards the well-being of people with disabilities. In this regard, funding might be more beneficial if it were fed directly from donors to DPOs. This would both eliminate the need to fund INGO staff and ensure that funding is used according to the DPOs’ needs.

While encouraging the disability movement, INGOs in Burkina Faso tend to *dominate* not only the disability movement but also the functioning of DPOs which are weak and remain dependent on the same INGOs. This is reminiscent of the observation by Drake (1997:644) that:

Charitable action and the evolution of government social policy has all too frequently reflected the hegemony of ‘nondisabled’ people.

Drake (1997:644) continues to say that ‘[*p*]erhaps disabled people’s organisations are now old enough (i.e. strong enough) to counter these kind of risks and may therefore be in a position to use the resources of ‘non-disabled’ allies’. However, as emerges from this research, while some DPOs in Burkina Faso might be ‘old’, they are not united in one strong disability movement. If, then, INGOs are to continue working with DPOs (something which people with disabilities do not reject), the onus lies with INGOs to support DPOs in becoming independent and self-sufficient. As Drake (1997:644) says, ‘[*i*]t is difficult to know where the proper balance lies here’. One way of finding a balance might lie in a suggestion made by one of the research participants, that is, of INGOs employing people with disabilities themselves in the projects they implement (Informal Conversation with Josiane, woman with physical disability, age 31). In this way, people with disabilities are directly involved in (and at different stages of) projects that affect them. From a study conducted by Flower and Wirz (2000:177) on European-based INGOs and grassroots DPOs, they found that while INGOs involve DPOs in the planning of their services and projects, this is mostly performed through sharing information with them, rather than ‘consulting them, including them in decision-making or supporting action initiated by’ the DPOs. Thus, as Kabzems and Chimedza (2002:149) assert, there is a ‘need to include persons with disabilities at all levels and stages of projects’. However, ‘[*i*]t remains rare for a person with a disability to be on the project payroll, whether in the capacity of consultant, accountant or tea lady’ (Kabzems & Chimedza 2002:149). Such observations, together with those which have been discussed throughout this article, highlight the importance of engaging with the lived experiences and voices of people with disabilities, and striving to involve them in all interventions affecting their lives. Such steps would endeavour to render development truly beneficial for people with disabilities in the Global South.

## Footnotes

1 *Les Amitiés Franco-Burkinabѐ* (The Franco-Burkinabe Friendships).

2 Handicap International has since changed its name to Humanity & Inclusion.

3 The term ‘grassroots’ is here used to differentiate these DPOs from umbrella DPOs. Definitions of these two types of DPOs are found in the ‘Research Findings’ section.

4 Information given to the researcher strictly in confidence, however, was not used in this article.

5 They are also referred to as organisations in French (including in Burkina Faso): *Organisations des Personnes Handicapées*.

6 The names of organisations have been omitted to ensure anonymity.

7 Such terms are ones used by the participants themselves.

8 Names of participants have been changed to ensure anonymity.

9 This denotes the age of the interviewee at the time of interview.

10 A centre where blind people used to be trained in furniture weaving. ‘*Noong Taaba*’ (in the Mooré language, the most commonly spoken language in Burkina Faso) means ‘We Love Each Other’.

11 These include both paid employment in the formal sector, or income-generating activities in the informal sector.

12 These are the researcher’s initials.

13 The interpreter and the author.
